# Hygiene procedures of trucks transporting live pigs: multi-assessment validation of a standardized C&D protocol

**DOI:** 10.3389/fvets.2026.1789484

**Published:** 2026-03-19

**Authors:** A. Perrucci, A. Magri, V. Cardana, S. Zoppi, C. Cossettini, A. Scollo

**Affiliations:** 1 Department of Veterinary Sciences, University of Turin, Turin, Italy; 2 Struttura s.r.l., Manerbio, Italy; 3 Istituto Zooprofilattico Sperimentale del Piemonte Liguria e Valle d’Aosta, Turin, Italy; 4 Chemifarma S.p.A., Forlì, Italy

**Keywords:** ATP, biosecurity, cleaning and disinfection, MRSA, practical recommendations, trucks

## Abstract

Inadequately cleaned transport vehicles can act as reservoirs for pathogens jeopardizing pigs’ health status. Although effective cleaning and disinfection (C&D) of live-pig transport trucks is required by law, there is still no universally accepted protocol for C&D of trucks. This study aimed to evaluate the effectiveness of a standardized hygiene protocol under field conditions and to provide practical guidance, thereby fostering harmonized hygiene procedures. Starting from current legislation for barn C&D, and refining it through available literature, a detailed standardized protocol for truck hygiene was developed. Each vehicle was divided into three functional sections: transport unit, boot-storage compartment, and driver’s cabin. The protocol was applied to 15 trial trucks transporting live pigs and compared with 23 market trucks, which served as controls. C&D was assessed through visual inspection, adenosine triphosphate (ATP) testing, and microbiological analyses of environmental samples, including total viable count (TVC) on all trucks. Samples were collected at the three functional sections of the trucks. Trial trucks achieved significantly higher visual scores than control trucks (82.10 ± 9.72 vs. 72.20 ± 7.48; *p* = 0.0018). The 80% cleanliness threshold required for ATP testing was achieved by nearly half of the trial trucks (46.7%) but by only one control truck (4.3%) (*p* = 0.0065). Microbiological results further highlighted the protocol effectiveness: all cleaned trial trucks showed low mean TVC values (<10 CFU/cm^2^ or a 3 log₁₀ CFU/cm^2^ reduction, as acceptable threshold). In contrast, only 33.3% of driver’s cabin swabs and 50.0% of cargo-area swabs from control trucks met this threshold, while none of the boot storage samples did (*p* = 0.0254). Bacteriological testing revealed MRSA in 100% of trial trucks before C&D, but in 0% after cleaning (*p* = 0.0079). Overall, the standardized protocol markedly improved the sanitary status of pig-transport vehicles. The combined use of different assessment methods proved valuable for identifying critical control points, particularly the boot-storage area, the most contaminated site. The protocol also showed strong potential for eliminating MRSA from trucks, contributing to reduce antimicrobial resistance transmission. These findings provide a replicable and field-ready model for improving C&D compliance and biosecurity across the swine transport sector.

## 1 Introduction

The current pig industry is heavily reliant on road transportation, with trucks moving between multiple farms, slaughterhouses, and loading zones, exposing them to pigs with different sanitary status. Wheels, ramps, and undercarriages can pick up fecal matter, urine, or bodily fluids and spread it across large geographic regions. As such, inadequately cleaned and disinfected trucks must be regarded as a major sanitary risk factor, since it can act both as a source of a disease introduction and a driver of pathogens propagation once an area becomes infected (1–3). Therefore, trucks must be considered an unacceptable biosecurity risk capable of triggering outbreaks at both the country and farm level (4, 5), especially under the current threatening ASF epidemiological scenario (2), although documented cases virus dissemination through trucks are rare (6). Since no vaccines against ASF are available, biosecurity is the only effective weapon against this pathogen (6), and truck hygiene has been mentioned as one of the key elements for ASF control and eradication (7–9).

In this framework, the cleaning and disinfection (C&D) procedures of vehicles used for transporting live pigs represent a critical control point (4). When properly performed after every transport, C&D can disrupt the contact network generated by truck movements between farms, thereby reducing indirect farm-to-farm contacts (4, 10, 11). From a broader perspective, improving the biosecurity of haulers and vehicles also contributes to combating antimicrobial resistance by limiting the spread of resistant bacteria among farms (12, 13). Ultimately, prioritizing preventive veterinary management strategies remains essential for driving the sustainability transformation of the livestock sector.

Indeed, C&D of vehicles transporting live animals is a legal requirement imposed by Animal Health Law (AHL) (14). According to literature, making C&D of transports mandatory by law is likely to be important for compliance, since the implementation of biosecurity measures varies among farmers and transport operators (15–18), but unfortunately it seems particularly difficult to implement only by legal requirement (19, 20). In Commission Delegated Regulation (EU) 2020/687 (9), supplementing the AHL and listing ASF as a category A disease, it is stated that trucks must be cleaned and disinfected “in accordance with the instructions or procedures provided for by the competent authority”. Therefore, the authorities from all the Member States are expected to provide a C&D protocol for the proper implementation of C&D trucks. Currently, authorities across the EU Member States issue guidelines for truck C&D; however, these are generally not legally established or officially recognized protocols. Moreover, in most cases, no methods are provided to authorities for verifying the proper implementation of these procedures. At the global level, legislation on the C&D of trucks transporting live pigs, mandatory in most cases, varies from country to country. Some countries have stricter regulations or more detailed guidelines (21–23), while others lack any formal framework.

Despite existing legal requirements for C&D of trucks transporting live pigs, this highly sensitive and very important task is often underestimated by the designated personnel because of these legal gaps or undefined procedures (5, 15). Kim et al. (24) reported that driver’s compliance with hygiene procedures alone is not sufficient to ensure satisfactory outcomes, highlighting the need for a detailed protocol with practical recommendations supported by an inspection and evaluation system. Even though no standardized or verified hygiene procedures are available, they are often implemented at private company level. At this point there is clearly the need to review the existing measures in order to achieve the best possible hygienic standards by optimizing the existing legal requirements and literature (3, 5). Additionally, sharing best practices and promoting knowledge exchange among countries can contribute to elevating biosecurity standards industry-wide (13).

Since the scientific community has not yet established universally accepted procedures or shared practical recommendations for truck C&D (5), this study aims to provide practical guidance for pig workers in their daily activities by validating the best possible standardized multi-phase protocol, detailed with practical and actionable recommendations for the C&D of trucks transporting live pigs.

## 2 Materials and methods

The instructions for C&D of barns present in the Italian Decree June 22nd, 2022 (25) and in the Spanish Royal Decree 638/2019 of November 8th (26) were the starting point for the development of the optimal truck hygiene protocol, which was further detailed based on the literature and existing regulations and guidelines. Subsequently, the developed C&D protocol was applied to 15 trial multi-level trucks transporting live pigs to identify and address possible shortcomings or practical challenges in its on-field application. To evaluate the effectiveness of the properly applied hygiene protocol, 23 market trucks were selected as control. These market trucks, owned by various swine producers and trucking companies, served as the control group as they did not follow any standardized C&D procedure, but rather applied their own cleaning practices based on current knowledge and experience. The 15 trial trucks and the 23 control trucks were selected through convenience sampling among multi-level vehicles transporting live pigs. Enrollment was consecutive and based on the daily operational schedule of the disinfection platform. Trial and control vehicles accessed the disinfection facility on different days during the study period. In all cases, the C&D procedures were carried out by the drivers themselves, who had been previously informed that the cleanliness of the vehicles would be evaluated upon completion. For the evaluation of the protocol, both trial and control trucks were assessed through visual inspection, adenosine triphosphate (ATP) environmental testing and microbiological analysis on environmental and air samples.

### 2.1 Development and application of the standardized C&D protocol

**Figure 1:**
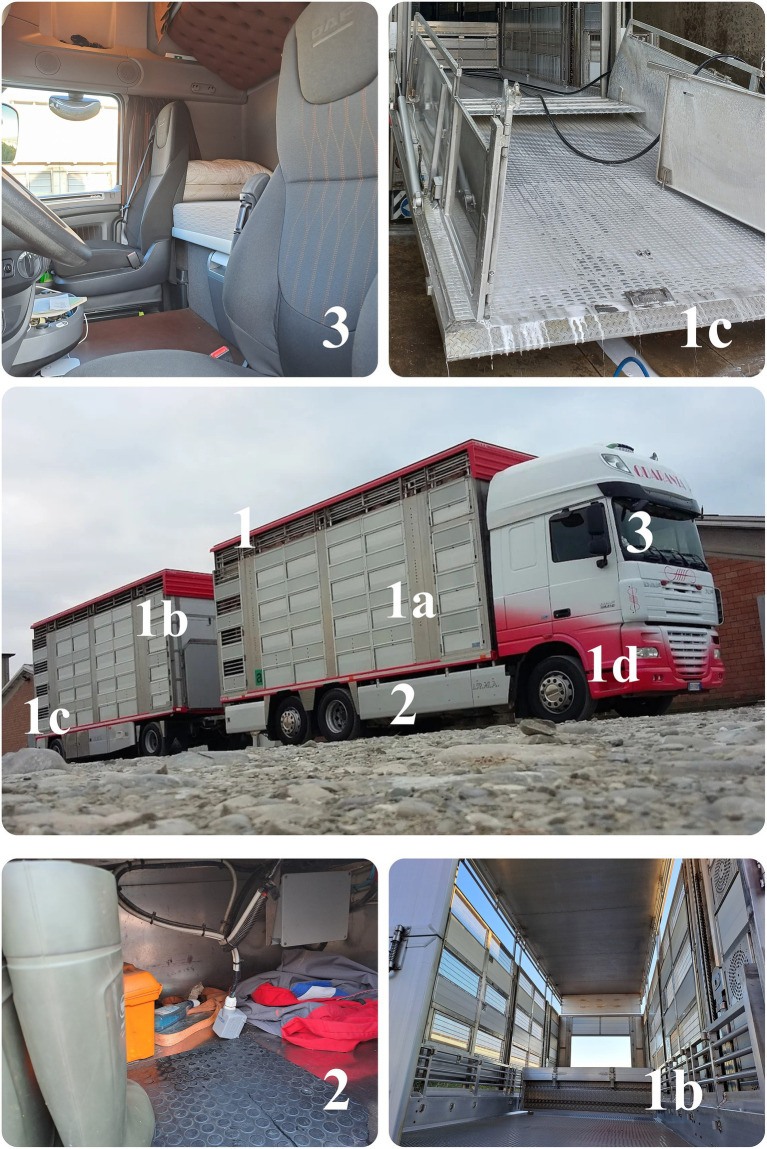
Schematic representation of the three sections of the C&D protocol: [1] transport unit, including [1a] the exterior and [1b] the interior of the livestock cargo compartment, [1c] the loading ramp, and [1d] the tractor; [2] boot storage; and [3] driver’s cabin.

To ensure any part from being neglected (7), the protocol was developed by dividing the truck into three sections, each dedicated to a specific part of the vehicle (Figure 1): [1] the transport unit, including both exterior and interior of the livestock cargo compartment, the loading ramp and the tractor, [2] the boot storage, and [3] the driver’s cabin. Before starting cleaning, all non-fixed objects in the transport unit and the boot storage (e.g., animal handling boards, shovels, and boots) had to be removed from the vehicle to be cleaned and disinfected separately at the end of the washing and disinfection process (27). In the event of potential vehicle contamination with a zoonotic pathogen, the C&D protocol described herein should necessarily be complemented by additional preventive measures targeting operator protection, in order to minimize the risk of transmission to humans. Such measures, which are strongly advised even in the absence of known zoonotic agents, should be developed *ad hoc* and are not included in the present description.

**Figure 2:**
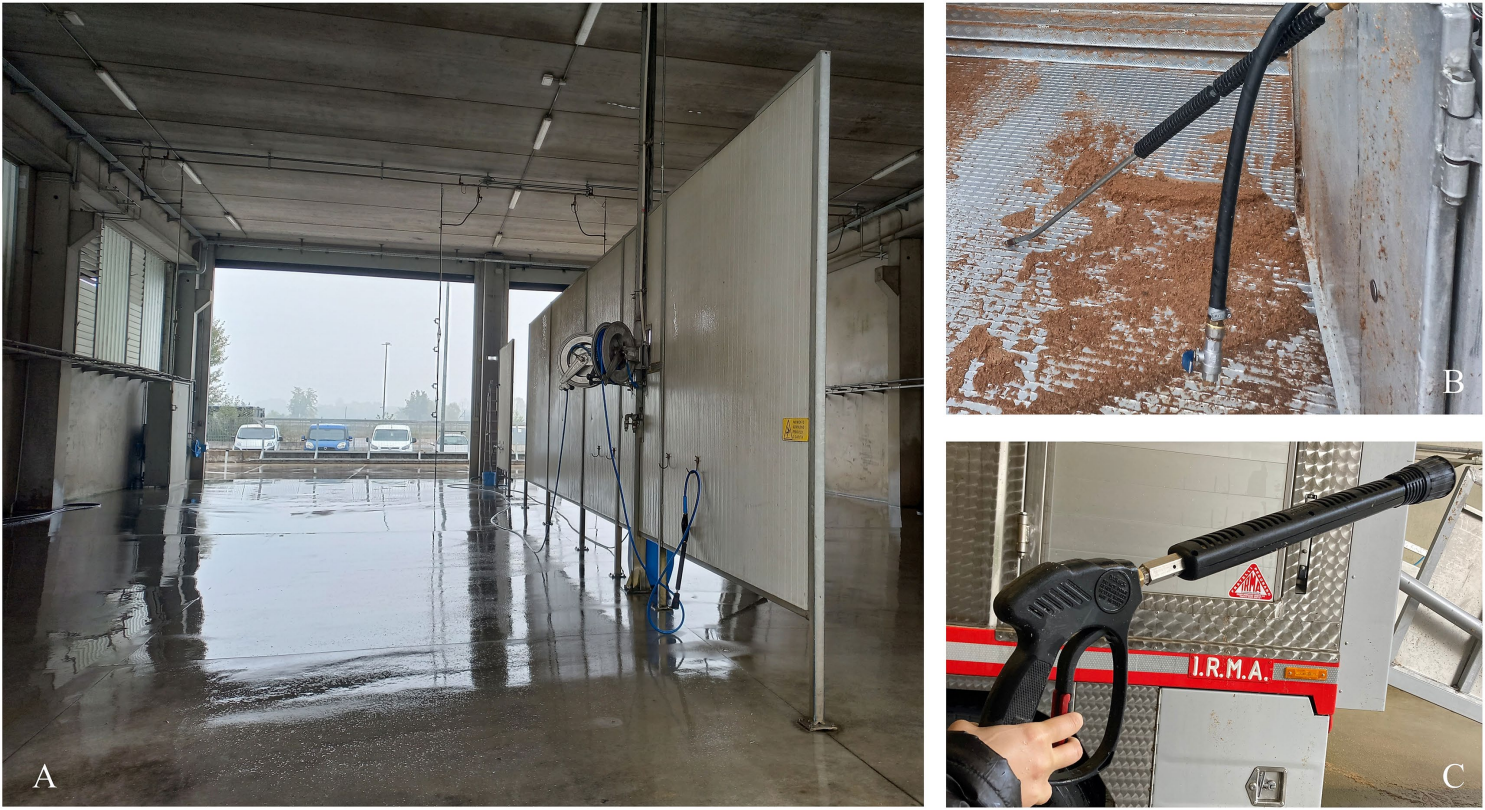
Photograph of the washing and disinfection bay with the equipment used for the trial trucks: **(A)** covered station with the disinfection arch visible at the far end, **(B)** low-pressure, high-flow water hose, and **(C)** foam gun.

For the correct execution of the C&D procedures, a dedicated and covered C&D 38 × 24 × 10 m station was required where operators could find all the essential equipment for carrying out the protocol (Figure 2), including a low-pressure and high-flow water hose (approx. 12 bar, water capacity 80–100 L/min), a high-pressure washer (approx. 100–120 bar, water capacity 10–15 L/min), a foam lance (approx. 180 bar) and a disinfection arch (5). The disinfection arch included two side manifolds 2 meters high and a recessed manifold in the bay floor, with a nozzle capacity of 8.8 L/s, using twenty-one nozzles. Eight nozzles were located on each of the two side manifolds, and five nozzles were installed in the floor manifold. The washing station had only ambient-temperature water available. Considering the importance of keeping the washing bay environment as clean as possible to avoid contaminating the “next truck” (23), the selected washing place had a concrete floor, impervious to liquids and sloped toward a drain for wastewater collection and was equipped with a hand-washing point. Additionally, the designated personnel (i.e., the driver of each truck) were equipped with waterproof protective clothing, boots, a helmet, a visor, and gloves (27).

#### 2.1.1 First section: transport unit

For the transport unit, hygiene procedures were applied in two phases: first, cleaning, which included pre-washing and washing (soaking and rinsing); and second, disinfection (28). The entire C&D procedure was structured into four areas, each represented by a specific component of the transport unit: the external surfaces of the animal compartment, the internal cargo area, the animal loading ramp, and the external surface of the tractor. This presenting order corresponds to the cleaning sequence followed by the personnel. The detailed procedures outlined below were implemented for each of the four designated areas of transport vehicle.

##### 2.1.1.1 Cleaning, first step: pre-washing

**Figure 3:**
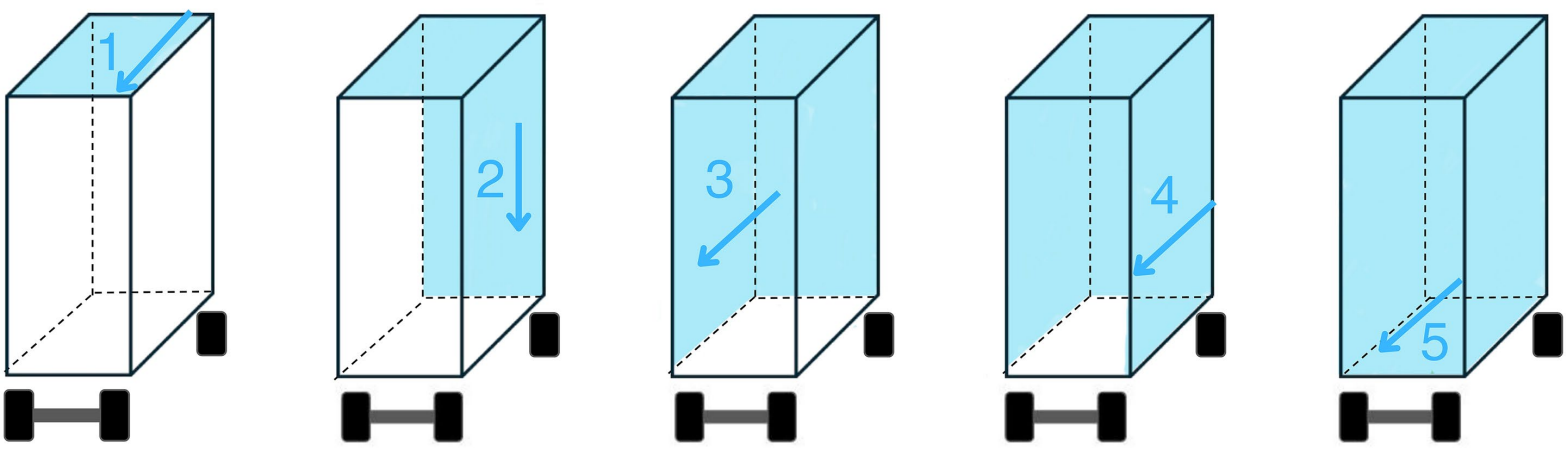
Strict cleaning order of surfaces from top to bottom: ceiling (1), rear wall (2), lateral walls (3, 4), and floor (5). Cleaning should always start from corners and perimeters, following a front-to-back flow from the vehicle’s perspective, as indicated by the arrows, to ensure no areas are missed by the operator.

**Figure 4:**
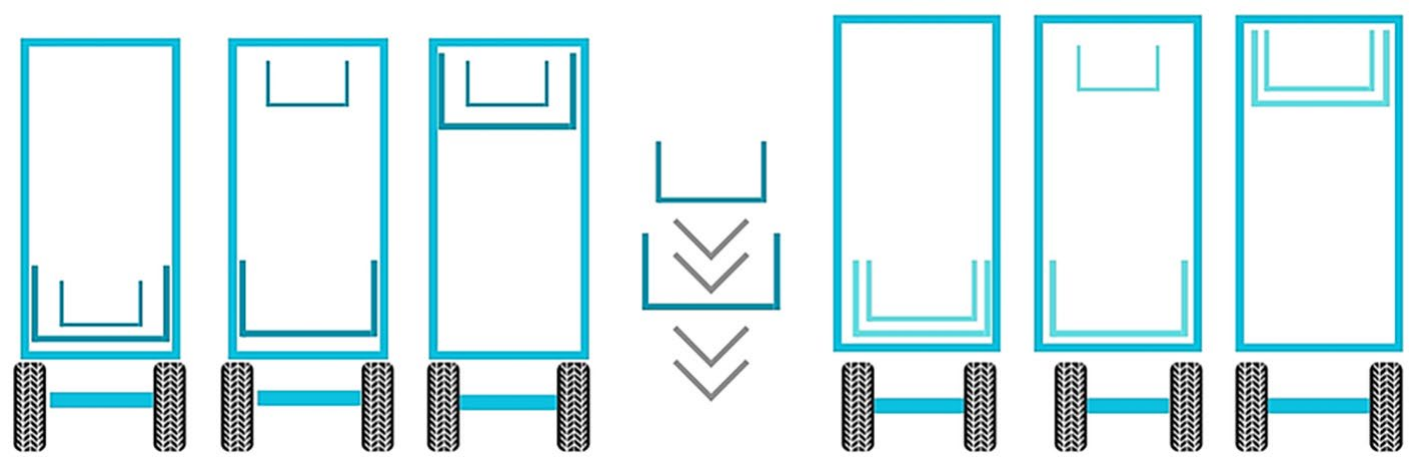
All internal floors were sloped downward to allow cleaning to start from the highest floor of the vehicle and proceed toward the lower areas, minimizing the risk of cross-contamination (21).

The first step was the pre-washing of all surfaces, aimed at removing gross debris. It consisted of an initial dry phase, followed by a wet phase using low-pressure water. The dry pre-washing phase consisted in manually removing as much solid debris as possible from horizontal surfaces, including manure and bedding, using a shovel or rake to scrape off debris from each floor of the truck. This first step involves the removal of highly variable amounts of solid material, depending not only on the quantity but also on its condition. During summer, for example, organic matter dries more quickly, making this phase more critical than it typically is in winter. For this reason, these factors also have a significant impact on the duration of this phase. According to literature (10, 29) this passage is highly effective at removing feces and other contamination better than using water alone. After, the wet pre-washing was performed by the operator through a low-pressure and high flow water hose (8), since it also prevents the raising of dust from the surfaces during washing, limiting the environmental spread of pathogens (27). Moreover, a high-pressure water blaster was not recommended because it would have been inefficient at moving large amounts of bedding or fecal material off the vehicle surfaces, as it would tend to spread the organic material inside the transport unit rather than rinsing it away (21–23). The water used was at the same temperature as the pipes (21). The wet pre-washing procedure had to be executed following a strict order on surfaces top-to-bottom (ceiling, walls, floors in this order, always starting from corners and perimeters, not to be forgotten by the operator) and front-to-back of the vehicle (Figure 3). Before starting the procedure, all internal floors were lowered, in this way the cleaning process was started from the highest level of the vehicle and proceeded towards the lower areas to avoid cross-contamination (21) (Figure 4). This sequence of steps ensured compliance with a workflow that minimized cross-contamination of areas already pre-washed, directing organic material to exit through the rear door of the truck, where the ramp was located, and onto drainage grates for proper disposal (21). During the pre-washing, emphasis was directed towards areas recognized as critical, e.g., any surface irregularities which could serve as reservoirs for pathogens (20, 30, 31).

##### 2.1.1.2 Cleaning, second step: washing

The washing phase included the application of the detergent by foaming and the rinsing; at the completion of this phase, the vehicle was free of any visible organic material, and ready for disinfection. The non-corrosive and potassium-hydroxide based detergent (Biosafe, Cid Lines, Unitec Srl, Milan, Italy) was an alkaline cleaning agent recommended for use on aluminum and stainless-steel surfaces, as it does not cause damage to these materials (8). It was used because it removes soil and organic materials reducing surface tension and increasing the penetrating ability of water, improving disinfectant’s ability to reach and destroy microorganisms within or beneath the organic material (8). The detergent was applied at 2% concentration using a foam gun (30) since it improves detergent efficiency, easier product application, as the operator could easily recognize the already treated surfaces from the not treated ones, and it allows to save detergent (23). Moreover, the use of foam ensures a longer contact time for the detergent to take effect, as it adheres better to surfaces and allows high or hard-to-reach areas to be treated more effectively (5). This effect is guaranteed by a properly mixed cleaning foam, which, when correctly applied to livestock housing surfaces, appears as a uniform, dense, and stable layer of white foam that adheres to both vertical and horizontal surfaces without immediate runoff, thereby ensuring effective organic matter loosening and microbial inactivation. This also allows the use of water at ambient temperature, rather than necessarily using hot water. It was the operator’s responsibility to ensure that no areas of the surface were left without foam: the operator’s attention through visual inspection played a key role, since they could repeat a passage of the procedure if they were not satisfied with the first application. In case of persistent organic matter, contaminated areas and corners were thoroughly brushed to avoid biofilm formation (23). The foam detergent had to be applied bottom-to-top with horizontal waving movements, since if it is applied top-to-bottom dirty foam detergent already applied on the top dripped onto the still clean surfaces below, and front-to-back, to avoid cross-contamination. Moreover, adhering to the contact time recommended by the detergent manufacturer was essential to ensure its effectiveness; failure to do so could compromise the overall cleaning outcome. During contact time, surfaces were monitored to make sure they did not dry off. If surfaces are drying, they should be sprayed with detergents again (8).

Thereafter, the foam was thoroughly rinsed using a high-pressure water hose; this was a critical passage, because any residues of organic matter or detergent can interfere with disinfection efficacy. It was the operator’s responsibility to perfectly rinse the surface, even getting very close to the surface with the high-pressure hose tip if necessary, and the strict order top-to-bottom, front-to-back starting from the highest floor was followed by the operator to minimize splashing onto previously rinsed areas, as explained above.

##### 2.1.1.3 Disinfection

A non-corrosive 2% glutaraldehyde (Glutarsan, Chemifarma S.p.A., Forlì, Italy) solution was used as the disinfectant. The choice was guided by the necessity of it being effective against a wide range of microbes, compatible with the selected detergent and non-corrosive to equipment and vehicle surfaces (8). The disinfectant was applied by wet nebulization from the nozzles of the external arch, strictly following the manufacturer’s indications concerning the solution preparation and the contact time. The truck, without being dried beforehand, was driven back and forth under the arch for a total time of 5 min, with the side windows open and the internal levels evenly spaced. The amount of disinfectant needed was 100 mL/m^3^ (32). After disinfection, trucks underwent an overnight downtime to allow for air-drying, enabled by consistently mild weather conditions, with temperatures never falling below 0 °C during the study. Drying is considered by literature a very important step for pathogens inactivation such as PRRSv (10, 29, 31), for preventing any disinfectant residue from being accidentally absorbed by the animals (27), and it guarantees adequate contact time to allow the disinfectant to exert its full efficacy.

##### 2.1.1.4 C&D specific practical recommendations

###### 2.1.1.4.1 Exterior surface of the truck

The first step of this protocol focused on the exterior surface of the truck and/or trailer, which was subjected to a double cleaning phase. First, the truck was correctly positioned in the C&D station with lateral windows and rear door closed. The operator started by cleaning the exterior surface using a low-pressure and high flow water hose and following the indications above presented. After, the foam application and the rinsing were performed twice, the first time with lateral windows open, and the second time closed. During this phase, particular attention was given to the wheels and fenders, as they can represent a distinct fomite that requires rigorous attention in order to achieve effective C&D (8). Attention was also paid to the ladder used by drivers to access the cabin. All wheels, fenders and ladder were cleaned a second time (foam detergent application followed by rinsing) at the end of the cleaning procedures of the entire transport unit.

###### 2.1.1.4.2 Inside cargo area of the livestock compartment

For each internal deck, the central divider, if present, was left raised so that it could be cleaned on both sides. Special attention was devoted to the dislodgement of organic matter from hard-to-clean areas, such as floor-wall junctions, internal partitions and sliding gates, drainage holes and floor drains ramp hinges, ventilation grilles and ramp hinges and joints, as these sites are particularly prone to the accumulation of debris and may serve as persistent reservoirs for microbial contamination.

###### 2.1.1.4.3 Loading ramp

The operator cleaned and washed first the internal surface of the loading ramp, opening and closing all of the side rails, secondly the external tailgate and the part beneath, where the license plate is located, after closing the ramp. Finally, the exterior part of the rear door was washed with foam and rinsed.

###### 2.1.1.4.4 Tractor unit

The tractor unit hygiene procedure, conducted after each transport, followed the instruction above presented for the trailer, but with slight changes. The cleaning step included a first passage using the low-pressure hose; afterwards, the detergent was applied by a detergent-soaked brush, and then it was rinsed by the high-pressure hose. The brush was equipped with a handle approximately 1.5 m long, allowing the operator to reach the upper part of the tractor unit. The washing procedure was repeated where the operator deemed appropriate based on visual evaluation.

#### 2.1.2 Second section: boot storage

Since boots are considered a high-risk medium for the transmission of infectious agents, it was deemed useful for the boot storage to be cleaned and disinfected separately from the rest of the truck (5), so that personnel could devote more attention and care to it. Boots and the boots container, if present, were cleaned and disinfected together with the equipment used for pigs handling. The cleaning steps are the same as those applied to the rest of the transport vehicle, as described above, and disinfection through nebulization could be carried out either manually using a disinfection lance or through automatic disinfectant dispensing systems that were directly installed inside the boot storage.

#### 2.1.3 Third section: driver’s cabin

The final step involved the cleaning and disinfection of the driver’s cabin, which was performed after each transport. The entire cabin was cleaned by the operator using compressed air (with particular attention to the steering wheel, dashboard, and gearshift), after all removable objects had been taken out, including the mats, which were also carefully shaken to eliminate debris. Subsequently, sanitization with a spray disinfectant (70% ethyl and isopropyl alcohol) was conducted on internal surfaces, the rearview mirror, the side mirrors and the dashboard glass.

### 2.2 Evaluation methods of the C&D protocol

On all trucks, visual evaluation and environmental samples collection were carried out by the same investigator, who wore single-use protective clothing, disposable shoe covers and gloves and changed them between each vehicle. All samples were collected within 30 min post-disinfection, ensuring uniform assessment conditions across all trucks. All samples (swabs and air samples) were stored at 4 °C until the analysis was performed within 48 h of sampling. Moreover, to evaluate the time required for the complete and correct execution of the developed C&D protocol, the timer was started as soon as the trucks reached the washing bay and stopped at the end of the disinfection process.

#### 2.2.1 Checklist for the visual evaluation

The visual evaluation was carried out at the end of the C&D process using a checklist based on the Australian Pork Industry Quality Assurance Program guidelines (33). The checklist covered the sections of the cleaning protocol proposed in this study: the transport unit—here divided into the exterior and interior of the livestock cargo compartment—the boot storage, and the driver’s cabin (detailed in Supplementary Table 1), for a total of 34 items, each rated on a 3-point scale. Scores ranging from 1 to 3 were assigned based on the extent of visible residual organic matter at each evaluated site. A score of 3 indicated a completely satisfactory site (no visible organic matter); a score of 2 was assigned when only traces of organic matter were present (covering less than 15% of the surface); and a score of 1 was given to unsatisfactory sites, those where more than 85% of the surface was contaminated, or where gross, highly evident contamination was observed, indicating substantial soiling rather than minor residue. Inspections were conducted immediately prior to environmental swabbing to avoid cross-contamination.

#### 2.2.2 ATP rapid test

ATP rapid tests (CleanTrace Surface ATP Test Swab UXL100, 3M, Neuss, Germany) were conducted following the methodology described in Scollo et al. (20). Values obtained were expressed as log₁₀ RLU/cm^2^, meaning that higher values correspond to a greater residual organic matter on surfaces. ATP was assessed in three sampling points: the interior cargo compartment, inside the boot storage, and in the driver’s cab (on driver’s and passenger’s seats, pedals, steering wheel, and gear shift). ATP bioluminometer testing was not performed prior to the application of the cleaning protocol (i.e., on soiled surfaces) due to the limited effectiveness of the instrument in accurately quantifying extremely high ATP levels typically found on dirty surfaces. Accordingly, ATP testing was not performed on swab samples collected from surfaces where visible organic residues remained (corresponding to a visual checklist score ≥2), due to the known unreliability of ATP measurements under such conditions. In fact, when surface contamination is excessive, ATP readings tend to reach saturation levels or become inconsistent, compromising their reliability for baseline assessment (34).

#### 2.2.3 Microbiological analysis

For microbiological analysis, environmental samples were collected from the same three sites selected for ATP testing, sampled before (dirty surfaces) and after (clean surfaces) the application of the C&D protocol, yielding six samples per truck. To collect samples from each surface, the area was wiped horizontally and vertically, covering 100 cm^2^ at each sampling site.

In order to obtain more quantitative information on the effectiveness of the C&D protocols, and due to the unreliability of ATP testing on heavily soiled surfaces, which therefore was not performed in this study (34), mesophilic aerobic total viable count (TVC) analysis was conducted on environmental samples. TVC is a widely used parameter for assessing surface cleanliness, as it reflects the total number of aerobic and facultative anaerobic microorganisms present (34). Additionally, molecular analyses were performed to detect Porcine reproductive and respiratory syndrome virus (PRRSV) and Porcine circovirus type 2 (PCV2), to further assess the presence of specific swine pathogens.

An additional, targeted assessment was conducted exclusively on the trial trucks to enable a more detailed evaluation of the C&D protocol. Specifically, environmental swabs were analyzed for the presence of methicillin-resistant *Staphylococcus aureus* (MRSA), and bacteriological air sampling was performed. The decision to include MRS screening was based on the pathogen’s known persistence in livestock environments and its relevance as sentinel microorganisms to monitor antibiotic resistance and a zoonotic agent with increasing public health concern (20).

Air samples were collected using the DUO SAS Super 360 (VWR Collection) from the inside cargo area of trial trucks before and after hygiene procedures. The aspiration pump operated at a total flow rate of 360 L min^−1^, providing an air flow of 180 L min^−1^. Standard 90 mm Petri dishes containing growth medium for bacteria were loaded into the sampler heads. The sampling heads were rinsed in 70% ethyl alcohol before each sampling. The air sampler was placed on a stool in the middle of the cargo area, with lateral windows closed. The sampling time was set in order to collect 550 L air volume (35).

##### 2.2.3.1 Bacteriological investigations

TVC was carried out on all the environmental samples following ISO standard procedure ISO 4833-1:2013 (36). Briefly, samples were homogenized and serially diluted from 10^−1^ to 10^−5^ dilutions in phosphate-buffered saline (PBS). Aliquots (100 μL) were plated in duplicate onto Plate Count Agar (PCA) using the pour plate method. Plates were incubated at 30 ± 1 °C for 72 ± 3 h, after which colony-forming units (CFU) were counted and expressed as CFU per swabbed area (CFU/cm^2^).

Exclusively for the trial trucks, the 10^−1^ dilution from the environmental samples was stored at −80 °C for 24 h. After pooling 200 μL from each sample, direct plating was performed on chromogenic media: Oxacillin Resistance Screening Agar Base (ORSAB, Oxoid, Wade Road Basingstoke, UK), used for the detection of methicillin-resistant *Staphylococcus aureus* (MRSA). The plates were incubated at 37 °C under aerobic conditions for 48 h (37).

Regarding air sampling, after the air collection, the plates were removed from the sampling heads of the DUO SAS and incubated at 37 °C for 48 h (35).

Subsequently, on all the plating and in case of colony growth, a colony count was performed, followed by identification through the selection of one colony per type, which was subcultured on Columbia Blood Agar (Oxoid, Wade Road Basingstoke, UK) and identified via MALDI-TOF (Bruker Daltonik GmbH, Bremen, Germany).

##### 2.2.3.2 Molecular diagnostics

DNA and RNA were extracted from environmental swabs diluted at 10^−1^ in PBS using the Molecular Biology Viral Nucleic Acid (DNA/RNA) Extraction Kit I (Thermo Fisher Scientific), following the manufacturer’s instructions. Quantitative and qualitative evaluation of extracted DNA was performed by spectrophotometric analysis. PRRS screening was conducted using VetMAX PRRSV 3.0 (Thermo Fisher Scientific), according to the manufacturer’s instructions. Primers for PCV2 DNA detection (CF8 5′ TAG GTT AGG GCT GTG GCC TT-3′ and CR8 5′ CCG CAC CTT CGG ATAT TAC TG-3′) were designed to amplify a fragment of 263 bp on the ORF2 region of PCV2 (38). For PCR reaction, 2 μL of extracted DNA were added to 48 μL of reaction mixture with final concentration of 1.25 mM MgCl2, 0.2 mM each dNTPs and 1 μM each primers using 2.5 U/reaction of AmpliTaq Gold™ DNA Polymerase (Thermo Fisher Scientific). DNA amplification was achieved by the following thermical profile: 35 cycles with denaturation at 95 °C/20 s, annealing at 55 °C/30s and extension at 72°/30 s followed by a final extension at 72 °C for 5 min.

### 2.3 Statistical analysis

For each of the three sections of the truck (four, considering the transport unit divided into the exterior and interior of the livestock cargo compartment) a total score from the visual evaluation was calculated by summing the individual item scores and then converting the result into a percentage of the maximum achievable score. Higher percentages indicated a higher level of visible cleanliness, and lower value a poor cleanliness status. Statistical analyses were performed with R version 4.5.1 (R Core Team, 2025). The Wilcoxon signed-rank test was used to estimate effectiveness of the developed C&D procedures comparing the results between trial and control trucks. Differences across the different sampling sites were analyzed by Kruskal–Wallis test for multiple comparisons, using Dunn’s method and Bonferroni’s correction. A linear mixed model was applied to assess the effects of the C&D procedures on TVC, considering group (trial vs. control), time (pre- vs. post-cleaning), and their interaction as fixed factors, and the truck as a random effect. Group comparisons for the proportions of frequencies were assessed using Fisher’s Exact test. The significance level was set at *p* < 0.05, with *p* < 0.01 as highly significant and *p* < 0.10 as a tendency.

## 3 Results

In trial trucks, the complete and correct execution of the developed C&D protocol required approximately two and a half hours for an articulated truck with a cargo area of about 130 m^2^. In contrast, for control trucks the C&D of an articulated vehicle at the slaughterhouse washing bay ranged from 20 min to 1 h, depending on how tight the driver’s working schedule was.

### 3.1 Checklist for the visual evaluation

The mean scores for the trial and control trucks across the sampling sites, as well as the overall mean, are presented in Table 1. All compartments showed a clear difference between the visual scores of trial and control trucks, except for a statistical tendency observed in the exterior of the cargo compartment, demonstrating an overall better visual cleanliness in trial compared to controls trucks. The four sections of the trial trucks’ visual checklist scores did not differ significantly (*p* > 0.05), indicating overall consistent cleanliness. In contrast, the four sections of the control trucks were significantly different (*p* = 0.0107). Specifically, Dunn’s *post-hoc* test with Bonferroni correction revealed a significant difference (*p* = 0.0153) in the percentage visual score between the boot storage (lowest score) and the interior cargo compartment (highest score). Nearly half of the trial trucks (7/15; 46.7%) obtained a visual cleanliness score exceeding 80% across all assessed compartments, whereas only a single truck (1/23; 4.3%) in the control group met this criterion (*p* = 0.0065).

**Table 1:** Mean (± SD) and *p*-values of the visual score (%) for each section of the checklist in trial and control trucks.

Sections	Trial trucks (*n* = 15)	Control trucks (*n* = 23)	*p* value
Transport unit			
Interior cargo	87.45 ± 11.36	78.03 ± 9.83^a^	0.0192
Exterior cargo	85.44 ± 8.39	76.57 16.53^ab^	0.0879
Boot storage	77.22 ± 18.22	63.40 ± 18.43^b^	0.030
Driver’s cabin	90.95 ± 10.33	70.76 ± 8.66^ab^	<0.0001
Total mean	82.10 ± 9.72	72.20 ± 7.48^ab^	0.0018

^a,b^Different letters mean a statistical difference among sections in control trucks.

### 3.2 ATP rapid test

**Figure 5:**
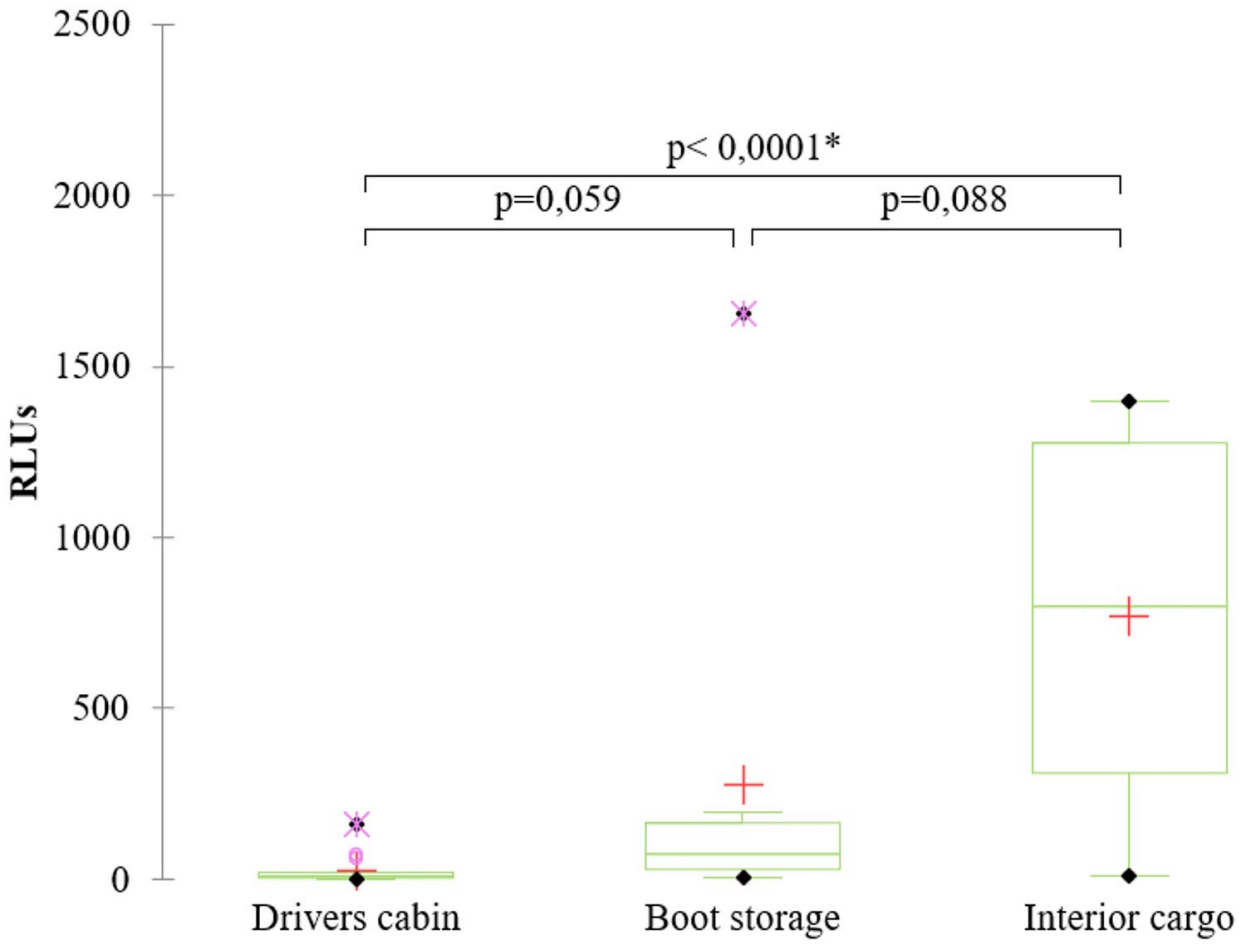
ATP test results (RLUs) for the three sampling sites in trial trucks: interior cargo compartment (*n* = 10; 770.8 ± 522.5 RLUs), boot storage (*n* = 8; 275.5 ± 561.2 RLUs), and the driver’s cabin (*n* = 14; 27.6 ± 45.1 RLUs).

Due to the presence of visible organic matter (i.e., visual checklist score ≤2), ATP testing could not be performed on a subset of sampled surfaces. Specifically, for the interior cargo compartment, ATP testing was not performed in 5 out of 15 trial trucks (33.3%), compared to 14 out of 23 control trucks (60.9%; *p* > 0.05). In the boot storage area, ATP testing was not possible in 7 out of 15 trial trucks (46.7%), versus 17 out of 23 trucks (73.9%; *p* > 0.05) in the control group. For the driver’s cabin, only 1 out of 15 trial trucks (6.7%) were excluded from ATP testing due to remaining organic matter, compared to 17 out of 23 control trucks (73.9%; *p* = 0.0002). Given the high proportion of samples excluded from ATP testing in the control group, ATP-related results are presented exclusively for the trial trucks (Figure 5): interior cargo compartment (*n* = 10) 770.8 ± 522.5 RLUs, boot storage (*n* = 8) 275.5 ± 561.2 RLUs and the driver’s cabin (*n* = 14) 27.6 ± 45.1 RLUs.

### 3.3 Microbiological analysis

**Figure 6:**
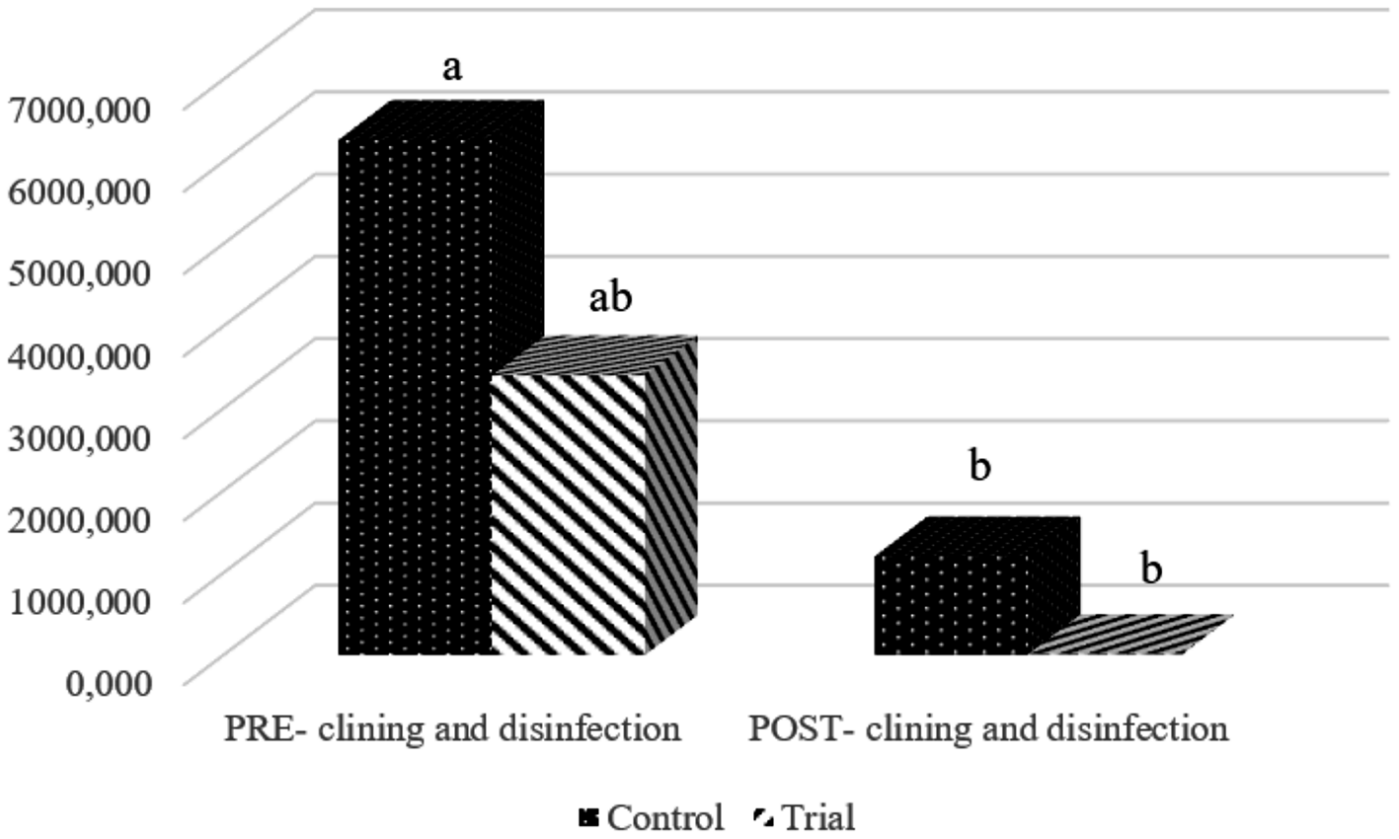
Mean total viable counts (TVC; CFU/cm^2^) before and after cleaning and disinfection procedures in trial and control trucks. ^a,b^Different letters indicate statistically significant differences (*p* < 0.05) between groups and timepoints.

The C&D procedures were effective in reducing TVC levels in both trial and control trucks (mean across all sampling points; *p* < 0.001). However, 100% of the cleaned trial trucks recorded low mean TVC values (<10 CFU/cm^2^; average bacterial load reduction of 2.06 ± 0.70 log₁₀.), whereas none of the control trucks (0%) achieved this threshold (*p* < 0.001). The control trucks did not even meet the hygiene threshold suggested by Böhm (39), who proposed a TVC value below 3 log₁₀ CFU/cm^2^ as a general target for a good disinfection in animal housing facilities. Focusing on the control trucks, the initial mean TVC value on dirty trucks was 6303.69 ± 4814.60 CFU/cm^2^ (versus 3399.0 ± 4562.97 CFU/cm^2^ for the trial group; *p* > 0.05). After the C&D procedures, control trucks showed an average of 1246.79 ± 3105.36 CFU/cm^2^, corresponding to an overall TVC reduction of approximately 1.26 ± 0.79 log₁₀. These differences are visually represented in Figure 6.

**Figure 7:**
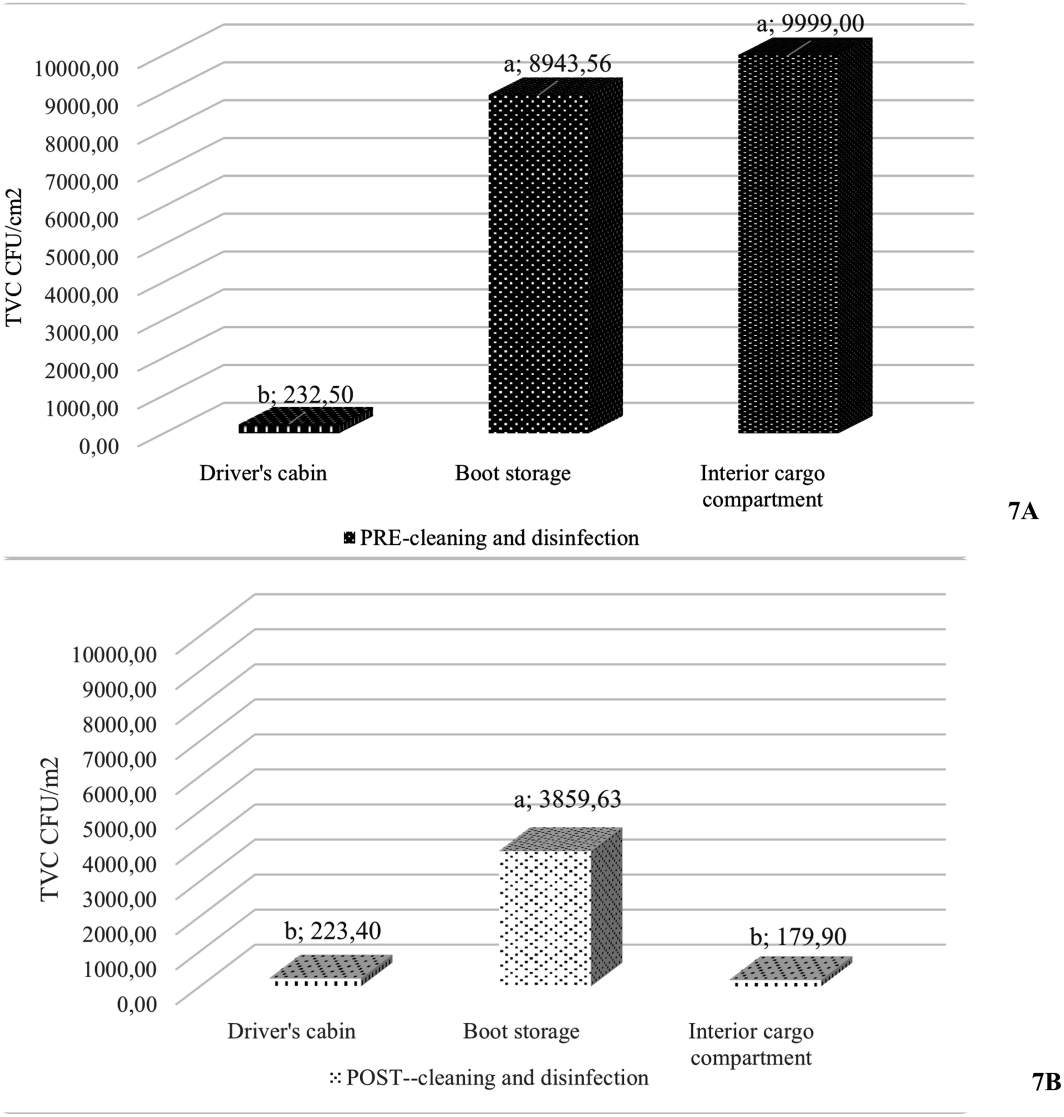
Mean total viable counts (TVC; CFU/cm^2^) of control trucks at each sampling site, measured before **(A)** and after **(B)** hygiene procedures. ^a,b^Different letters indicate statistically significant differences (*p* < 0.05) between sampling points before hygiene procedures.

Due to the lack of variability in post-cleaning TVC levels among trial trucks, the following analysis focuses on the detailed results from the different sampling points in control trucks only (Figure 7). Only 33.3% of the swabs from the driver’s cab and 50.0% from the interior cargo compartment reached the acceptable threshold of < 10 CFU/cm^2^, nor a 3 log₁₀ CFU/cm^2^ reduction (39). No samples from the boot storage met this threshold. In the dirty environment, TVC values differed significantly among the three sampling sites (*χ*^2^ = 24.072; *p* < 0.001): the driver’s cabin had significantly lower TVC levels compared to both the boot storage and the interior cargo compartment (*p* < 0.0001). However, C&D were effective in reducing TVC in the interior cargo compartment, achieving the greatest reduction (2.40 ± 0.77 log₁₀), and raising its cleanliness level to that of the driver’s cabin (*p* = 0.0001). The boot storage remained the most contaminated site after C&D (*p* = 0.0254; when compared to the interior cargo compartment: *p* = 0.0150; to the driver’s cab: *p* = 0.0453), although it showed a modest reduction in TVC levels (1.02 ± 0.91 log₁₀).

All environmental samples tested negative for both PRRSV and PCV2, in both trial and control trucks.

Bacteriological analysis from environmental samples of trial trucks revealed MRSA in all pooled swabs collected before C&D of trial trucks (100% prevalence), whereas no MRSA was detected in clean swabs (0% prevalence; *p* = 0.0079). Regarding air samples, the SAS system detected methicillin-resistant staphylococci in two dirty trucks (13.3%), and the C&D procedures successfully eliminated them in both cases. In the first truck, MRSA was identified, along with the presence of non-resistant *S. sciuri;* in the second, methicillin resistance was detected in *S. borealis.* Additionally, *S. hyicus* (non-resistant) was isolated in the third truck. *Klebsiella pneumoniae* was detected in the dirty environment in samples from two dirty trucks (13.3%), and *Streptococcus parasanguinis* was detected once. Other environmental bacteria were detected before C&D: *Bacillus* spp. (33.3%; *B. subtilis*, *B. cereus, B. megaterium, B. pumilus*), *Aerococcus viridans* (26.6%)*, Acinetobacter towneri* (13.3%), and once *Stenotrophomonas maltophilia*, *Micrococcus luteus*, *Rothia nasimurium*, and *Cytobacillus horneckiae*. After C&D procedures, air samples tested negative unless for *Bacillus* spp. (13.3%) and *Aerococcus viridans* (once). Unexpectedly, one truck was negative for infectious agents before C&D but tested positive for *Escherichia coli* and *Streptococcus* spp. in the clean environment.

## 4 Discussion

The C&D protocol presented in this study provides practical, standardized procedures for personnel responsible for C&D of trucks transporting live pigs, aiming to improve sanitary conditions and minimize pathogen spread. Cleaning is defined as the physical removal of dust and debris using water, with or without detergent and mechanical action, until surfaces are visibly clean (40); when properly performed, it can reduce contamination by up to 90% (27). Disinfection aims to eliminate as many microorganisms as possible, reducing the risk of infection and cross-contamination (41). Routine C&D therefore represents a combined strategy, with disinfection effectiveness heavily dependent on thorough cleaning, as residual organic matter can impair disinfectant activity. Despite C&D of vehicles transporting live pigs being a legal requirement (14) and playing a pivotal role in biosecurity, low compliance with lorries C&D is largely due to the absence of a clear standard operating protocol (5) and the lack of routine post C&D verification procedures. Moreover, only a few studies focused on the identification of areas that are consistently more difficult to sanitize on livestock trucks (42, 43), as also reported at the farm level (20, 44), even though knowledge of such weak points in C&D procedures is fundamental for improving biosecurity. Establishing a clear and comprehensive C&D protocol is essential, as it enables the implementation of training programs on best practices, thereby improving compliance with biosecurity measures (13, 20). The aim of the present study was therefore to address this gap by providing practical guidance for workers in their daily activities, through the development of a standardized and comprehensive multi-phase protocol which includes actionable recommendations ensuring C&D of trucks transporting live pigs.

The C&D protocol developed in this study was designed to be with a compartmentalized approach targeting four key areas of the truck: exterior, interior cargo, boot storage, and driver’s cabin. Its effectiveness was evaluated in 15 trucks and compared to 23 control vehicles lacking standardized procedures. A combination of assessment methods was applied across all compartments, including driver-related areas often neglected during routine cleaning, providing a robust and comprehensive evaluation framework (5, 15, 16, 34).

The first evaluation method applied was visual inspection, consisting in the individuation of visible residual organic matter on clean surfaces by an inspector. It is a quick, simple and cost-effective method that can be easily used as a first-line screening test to assess the efficacy of C&D procedures (34, 42, 43). This was confirmed in the present study, where visual inspection proved effective in demonstrating that the studied protocol improved cleanliness in trial trucks compared to control ones. Indeed, trial trucks scored high (mean score 82.10 ± 9.72%), in contrast with control trucks which registered lower scores in all four sections of the checklist (72.20 ± 7.48%). By setting the 80% threshold as the minimum acceptable score for visual cleanliness, visual inspection was able to more clearly highlight the difference between trial operators who followed a standardized C&D protocol and those who cleaned without specific guidance (control). Nearly half of the trial trucks (46.7%) exceeded the 80% threshold, whereas only one truck in the control group (4.3%) met this criterion (*p* = 0.0065). These results underline the importance of using visual scoring as an initial screening step, as failure to meet a sufficient cleanliness score may render further testing steps unnecessary. It is important to emphasize that differences observed between trial and control trucks cannot be attributed to a lack of awareness among the control truck operators regarding the post-cleaning evaluation, as all drivers had been equally informed beforehand about the assessment procedures. Moreover, visual inspection revealed that the application of a structured protocol, such as the one proposed in this study, helped to minimize differences between the various compartments assessed in trial trucks. In contrast, control trucks showed more marked variability (*p* = 0.0107), with certain areas that were particularly challenging for C&D, often because they are marginal and easily overlooked. A notable example is the boot storage, which drivers use at every load, but which consistently appeared as the dirtiest area.

In summary, the quick and cost-free identification of clean and dirty areas after C&D can be achieved through the routine use of visual checklists, which can be easily performed on-site by someone who is adequately trained, including haulers (45). Moreover, regularly conducting and recording such visual checks increases drivers’ and transport companies’ compliance with hygiene protocols (5). Nevertheless, this test remains superficial, as it cannot detect bacteria, viruses, or chemical residues that may still be present even when a truck appears clean (46). In addition, this test is highly subjective, owing to the lack of standardized definitions for ‘clean’ and ‘dirty’ (44), and it is prone to human error, since the perception of a surface’s cleanliness is strongly influenced by several factors, such as lighting conditions, surface shape, materials, and structural aspects that make difficult to visualize certain areas, which, if not inspected, may remain unclean (44). This is particularly likely on livestock trucks, whose complex structures, such as grates, fans, drinkers, and moving components can create hidden areas that are difficult to access and may remain uncleaned during standard C&D procedures. Furthermore, tight schedules in livestock transport can lead to rushed inspections or manipulation of checklists to meet deadlines (5), becoming a mere bureaucratic exercise, with operators performing superficial inspections without ensuring actual cleanliness.

### 4.1 Objective C&D assessment methods: ATP and TVC

Therefore, despite regular training of inspectors to support greater objectivity and accuracy in visual evaluations, using ATP testing offers several advantages. ATP levels could serve as a real-time quantitative biomarker to verify the effectiveness of sanitation procedures, unlike visual assessments, which remain subjective. In addition, incorporating ATP monitoring may help increase the engagement and accountability of personnel responsible for C&D protocols (20, 40).

As previously mentioned, by setting the 80% threshold as the minimum acceptable score for visual cleanliness, only one truck in the control group met this criterion, rendering further testing steps unnecessary. Therefore, ATP testing was conducted exclusively on the trial trucks. While visual inspection revealed minimal differences between the various compartments assessed in trial trucks, ATP testing identified the driver’s cabin as the cleanest area, followed by boot storage and the interior cargo compartment. Both the driver’s cabin and the boot storage of trial trucks showed ATP values lower than the threshold identified by Heinemann et al. (44) for pig fattening units, who proposed >500 RLU as indicative of inadequate cleanliness of pen surfaces. However, this marked difference with the interior cargo compartment was not reflected in the microbiological analysis of TVC levels. In fact, TVC levels in the interior cargo of trial trucks consistently remained extremely low (<10 CFU/cm^2^). Therefore, it cannot be excluded that the higher ATP values recorded in this compartment may have been influenced by residual surface moisture compared to the driver-accessed areas. Moreover, the presence of disinfectant residues that were not fully rinsed off in the interior cargo might have interfered with ATP analysis across all measurements (34, 47).

Considering the inability to evaluate ATP results from control market trucks, more specific and evidence-based feedback was needed through microbiological analysis. In order to have quantitative information on the effectiveness of C&D protocols, although less specific pathogen-targeted identification, TVC was performed on environmental samples (34). Swabs were preferred over agar contact plates, as they are more suitable for evaluating non-flat surfaces, such as those present in trucks transporting live pigs. Results were consistent with the visual score and ATP measurements: after applying the proposed protocol, the TVC of all trial trucks was reduced to the very low threshold of <10 CFU/cm^2^. In contrast, none of the control trucks reached that threshold, nor did they meet the second criterion for effectiveness (a 3 log₁₀ CFU/cm^2^ reduction) as suggested by Böhm (39), although the numerical reduction in CFU/cm^2^ from pre- to post-C&D was statistically significant. This indicates that while the cleaning procedures applied to control trucks were quite effective in reducing bacterial loads, they were not sufficient to ensure an adequate final state of cleanliness and disinfection.

However, a more detailed analysis of the different sampling sites revealed that this observation occurred mostly in the boot storage, which showed the most evident insufficiency in bacterial load reduction following C&D procedures—only 1.02 ± 0.91 log₁₀. Hence, the boot storage area remains the most neglected spot during C&D by haulers across all the assessment methods, consistently representing the most contaminated area of the trucks and a high-risk site for disease spread. Evidence specifically addressing this issue in the swine sector is scarce. To date, the available literature mainly refers to other livestock systems. In this context, studies investigating hygiene risks in driver zones reported that boot storage and boots in trucks were rarely disinfected, posing a serious biosecurity gap (5, 15, 16). In particular, Duarte et al. (16) reported that, in a survey of 81 cattle haulers, only one carried out complete C&D of their boots. Considering that pathogen transfer can occur from the boot compartment to the cabin, and then onto other farm locations, results of this study highlight the serious risk that many drivers may have reused their boots without properly disinfecting the storage compartment, despite efforts focused on cleaning the livestock areas. Therefore, market trucks pose a high risk for pathogen dissemination, not only during disease outbreaks like ASF (3), but also under normal conditions due to tight schedules and regulatory limits on drivers’ working hours (48), which leave less time for hygiene procedures.

The driver’s cabin was the cleanest area after C&D, consistent with the ATP values observed in the trial trucks. However, in the case of TVC analysis in control trucks, where both pre- and post-C&D samples were available, it emerged that the driver’s cabin already had low bacterial loads prior to cleaning, in contrast to the areas in direct contact with the animals and their waste. Drivers tended to focus more on the interior cargo compartment during the hygiene process, resulting in the greatest reduction in bacterial load (2.40 log_10_). Among specific pathogens investigated during the study, PRRSV and PCV2 were never detected. The hypothesis is that selected trucks moved from farms in a sanitary stability and were not facing any outbreaks.

### 4.2 Trucks C&D effectiveness against MRSA

When an additional, targeted assessment was conducted exclusively on the trial trucks to enable a more detailed evaluation of the C&D protocol, it was possible to verify that MRSA—initially present in all trucks before C&D—had been effectively eliminated from all vehicles following the procedures, as confirmed by both environmental and air samples. This result supports the effectiveness of the protocol proposed in the present study, and contrasts with reports in the literature on commonly implemented on-farm C&D procedures (20, 49, 50), where MRSA is often not completely eliminated from the environment.

The prevalence of MRSA observed in trial trucks in this study (100%) was higher than those reported in Italian pig farms by 0% Bonvegna et al. (51) and 22.5% Scollo et al. (20), and it also exceeded values reported in other European countries (6.5–36.3%), in Belgium and Switzerland, respectively (52, 53). The absence of such persistence in transport vehicles following the present protocol is therefore encouraging, and several hypotheses may explain this phenomenon. For instance, Schmithausen et al. (54) successfully decontaminated pig farms from MRSA by including complete depopulation in their protocol, like what occurs in vehicles, where trucks follow an all-in all-out flow and C&D is carried out in the absence of animals. This differs from some farm settings, where C&D is sometimes performed while pigs remain in adjacent barns or even adjacent pens. Since MRSA can be transmitted via air and dust and emitted through ventilation systems into the surrounding environment (55), recontamination in vehicles may be minimized by implementing strict biosecurity protocols that control the movement and separation of clean and dirty trucks at the washing bay. This risk is further supported by findings from the present study, in which MRSA, along with other pathogens increasingly recognized as public health threats, such as *Klebsiella pneumoniae* and *Streptococcus parasanguinis* (56, 57), was detected in air samples collected using a Surface Air System (SAS) sampler prior to C&D procedures. Interestingly, one trial truck air sample tested negative for infectious agents prior to cleaning but was found to be contaminated with *Escherichia coli* and *Streptococcus* spp. after the C&D procedures. This unexpected result suggests the possibility of post-cleaning recontamination, potentially linked to environmental exposure during the drying phase, especially when trucks are parked near contaminated areas, including barns or lairage facilities.

This observation underscores the importance not only of effective cleaning protocols, but also of environmental biosecurity measures following sanitation, to avoid compromising the effectiveness of C&D (58–60). In Bae et al. (58) a significant reduction in airborne microbial load was detected after air disinfection in veterinary hospitals, improving environmental hygiene which plays a critical role in infection prevention.

In addition to major pathogens, several environmental bacteria, such as *Bacillus* spp., *Aerococcus viridans*, and *Acinetobacter towneri*, were detected in pre-C&D air samples, reflecting typical environmental contamination (58). After C&D, only *Bacillus* spp. (13.3%) and *A. viridans* (once) persisted, confirming the importance of proper drying and strict spatial separation of cleaned vehicles to prevent recontamination from the surrounding environment. On farms, recontamination frequently occurs also through footwear, clothing, or cross-contamination from personnel (49). These risks are more easily controlled in trucks due to the designated parking areas where vehicles remain during C&D, which facilitate hygiene operations and allow for effective removal of waste and dirt through drainage systems.

The presence of MRSA in 100% of the trial trucks prior to C&D highlights the potential role of transport vehicles as a critical point of contamination in the swine production chain. Broens et al. (59) reported that pigs testing negative for MRSA at the farm can become positive during transport to the abattoir or while being held in lairage areas. This was attributed to environmental characteristics, such as rough concrete floors, which hinder effective disinfection and allow microorganisms to persist. The results of the present study strengthen this hypothesis, as the detection of MRSA in all trucks before cleaning confirms the high microbial burden in livestock compartments. Importantly, the C&D protocol applied here was able to eliminate MRSA entirely from all trial vehicles.

The high prevalence of MRSA even after C&D has been highlighted as a critical One Health issue (61), since MRSA is considered an indicator organism for the presence of antibiotic-resistant bacteria for both animals and humans (54). In this context, while the transmission of MRSA from truck drivers to pigs has not been conclusively demonstrated (59), available data suggest that drivers are more likely to be victims of occupational exposure than sources of infection. Studies in pig and poultry abattoirs have reported MRSA prevalences ranging from 22 to 40% among lorry drivers (62–64), underscoring the importance of robust hygiene protocols, not only to limit environmental MRSA loads and protect animal health, but also to safeguard the health of transport workers operating in high-risk settings.

Searching for the best application of a C&D protocol, the proper selection of disinfectants should not be overlooked, as their documented efficacy against priority swine pathogens, including African swine fever virus (8), as well as their ability to act against bacterial biofilms, can ultimately determine the success of C&D. MRSA, for example, is a biofilm-producing pathogen, recognized for its persistence and capacity to cause chronic infections, including in hospitals, where biofilm formation confers additional resistance to C&D (65). However, the available literature indicates that biofilm formation represents only one of several mechanisms potentially involved in MRSA persistence. MRSA persistence in livestock production systems is widely acknowledged to be multifactorial, resulting from the interaction between microbial characteristics, environmental contamination, animal-related factors, and management practices. In swine production, Merialdi et al. (50) demonstrated a preventive effect of C&D procedures on environmental contamination, although this effect was not constant across productive phases and was more pronounced in farrowing crates than in later stages. Longitudinal studies (66, 67) have shown that MRSA nasal carriage dynamics vary markedly with age, with differences in prevalence among herds largely attributed to management practices, while the role of the environment has been recognized as contributory and deserving further investigation.

The time required for the complete and proper execution of the developed hygiene protocol can be considered among the factors contributing to the improved sanitary status of the trial trucks, as it is longer than the average time recorded for control trucks at the slaughterhouse. Indeed, time constraints are frequently reported as a reason for haulers’ non-compliance with truck C&D procedures (5, 15, 68). While a duration of two and a half hours may be feasible for trucks transporting live pigs, it is hardly compatible with the slaughterhouse’s tight routine. Nevertheless, this protocol could serve as a starting point for improving hygiene management in market trucks, tailoring it to fit the slaughterhouse’s tight schedule while still ensuring an adequate sanitary level.

A limitation of this study is that the trial and control trucks did not belong to the same production chain and therefore transported different groups of pigs, which may have varied in terms of age, origin and health status. These differences could have influenced the baseline microbial load, particularly in compartments such as the interior cargo area. However, it is important to note that the aim of the study was not to directly compare performance between different fleets, but rather to demonstrate the effectiveness of the standardized C&D protocol applied to the trial trucks. The control group served as a contextual benchmark to support the observed outcomes in the trial group, rather than as a strictly matched comparator.

To further improve the proposed protocol, it is recommended to ensure that surfaces are completely dry before applying the disinfectant, as excessive water accumulation inside the vehicle can dilute the disinfectant and compromise its effectiveness, while increased residual moisture may also favor bacterial survival and proliferation (21). In addition, the improved protocol should also include cleaning both underneath and on top of the truck. Further research should focus on the validation of this C&D protocol in the field, and on individual ATP threshold and critical limits. To ensure the protocol’s effectiveness and long-term adoption, it should ideally be supported by regular training and reinforced through oversight by competent authorities or independent control bodies. To improve biosecurity of trucks, other strategies may be used, such as rerouting, as proposed by Galvis et al. (69). Finally, the authors emphasize that improving truck hygiene does not justify non-compliance with haulers’ biosecurity measures. Although the responsibility of the truck C&D lies with the animal transport driver (5), the widespread use of contract livestock haulers (instead of farmed-owned transport) hinders the enforcement and monitoring of biosecurity compliance (3). Haulers must remain aware of proper biosecurity practices on farms to avoid undermining the efforts invested in C&D.

## 5 Conclusion

This study demonstrates that the implementation of a standardized C&D protocol for livestock trucks transporting live pigs significantly improved sanitary status of trucks. Its availability represents a crucial step forward for the livestock transport sector, as it provides operators with practical guidance that should be applied routinely after each transport to minimize the risk of disease spreading via transport trucks. The application of visual inspection, ATP testing, and microbiological analyses provided a comprehensive assessment of cleanliness, highlighting critical control points such as the boot storage, which showed the highest residual contamination in control vehicles. Notably, the developed procedure also showed promising results in reducing MRSA contamination in pig trucks compared to the commonly applied farm-level practices. However, it should be emphasized that pigs represent the primary reservoir of MRSA, acting as nasal carriers, and that C&D procedures cannot address animal colonization. In this context, C&D primarily contributes to limiting environmental contamination within transport vehicles and may be considered a useful indicator of the overall effectiveness and safety of hygiene practices applied by operators, rather than a direct strategy for pathogen elimination. These findings underline the importance of adopting structured hygiene protocols, not only to enhance animal health through biosecurity, but also to reduce occupational exposure risks for drivers and prevent the spread of antimicrobial-resistant bacteria within the production chain. The practical approach and detailed procedures proposed in this study offer a replicable and field-ready model for improving C&D compliance and biosecurity across the swine transport sector.

## Data Availability

The raw data supporting the conclusions of this article will be made available by the authors, without undue reservation.
