# Correction: Hygiene procedures of trucks transporting live pigs: multi-assessment validation of a standardized C&D protocol

**DOI:** 10.3389/fvets.2026.1868848

**Published:** 2026-05-21

**Authors:** A. Perrucci, A. Magri, V. Cardana, S. Zoppi, C. Cossettini, A. Scollo

**Affiliations:** 1 Department of Veterinary Sciences, University of Turin, Turin, Italy; 2 Struttura s.r.l., Manerbio, Italy; 3 Istituto Zooprofilattico Sperimentale del Piemonte Liguria e Valle d'Aosta, Turin, Italy; 4 Chemifarma S.p.A., Forlì, Italy

**Keywords:** ATP, biosecurity, cleaning and disinfection, MRSA, practical recommendations, trucks

In the published article, references were numbered incorrectly starting from reference number 61 onwards. In section **4.2 Trucks C&D effectiveness against MRSA**, paragraphs 6–10, incorrect numbers were assigned to the in-text citations for these references, as per the content below:

“The high prevalence of MRSA even after C&D has been highlighted as a critical One Health issue (60), since MRSA is considered an indicator organism for the presence of antibiotic-resistant bacteria for both animals and humans (54). In this context, while the transmission of MRSA from truck drivers to pigs has not been conclusively demonstrated (59), available data suggest that drivers are more likely to be victims of occupational exposure than sources of infection. Studies in pig and poultry abattoirs have reported MRSA prevalences ranging from 22 to 40% among lorry drivers (61–63), underscoring the importance of robust hygiene protocols, not only to limit environmental MRSA loads and protect animal health, but also to safeguard the health of transport workers operating in high-risk settings.

Searching for the best application of a C&D protocol, the proper selection of disinfectants should not be overlooked, as their documented efficacy against priority swine pathogens, including African swine fever virus (8), as well as their ability to act against bacterial biofilms, can ultimately determine the success of C&D. MRSA, for example, is a biofilm-producing pathogen, recognized for its persistence and capacity to cause chronic infections, including in hospitals, where biofilm formation confers additional resistance to C&D (64). However, the available literature indicates that biofilm formation represents only one of several mechanisms potentially involved in MRSA persistence. MRSA persistence in livestock production systems is widely acknowledged to be multifactorial, resulting from the interaction between microbial characteristics, environmental contamination, animal-related factors, and management practices. In swine production, Merialdi et al. (50) demonstrated a preventive effect of C&D procedures on environmental contamination, although this effect was not constant across productive phases and was more pronounced in farrowing crates than in later stages. Longitudinal studies (66, 67) have shown that MRSA nasal carriage dynamics vary markedly with age, with differences in prevalence among herds largely attributed to management practices, while the role of the environment has been recognized as contributory and deserving further investigation.

The time required for the complete and proper execution of the developed hygiene protocol can be considered among the factors contributing to the improved sanitary status of the trial trucks, as it is longer than the average time recorded for control trucks at the slaughterhouse. Indeed, time constraints are frequently reported as a reason for haulers' non-compliance with truck C&D procedures (5, 15, 68). While a duration of two and a half hours may be feasible for trucks transporting live pigs, it is hardly compatible with the slaughterhouse's tight routine. Nevertheless, this protocol could serve as a starting point for improving hygiene management in market trucks, tailoring it to fit the slaughterhouse's tight schedule while still ensuring an adequate sanitary level.

A limitation of this study is that the trial and control trucks did not belong to the same production chain and therefore transported different groups of pigs, which may have varied in terms of age, origin and health status. These differences could have influenced the baseline microbial load, particularly in compartments such as the interior cargo area. However, it is important to note that the aim of the study was not to directly compare performance between different fleets, but rather to demonstrate the effectiveness of the standardized C&D protocol applied to the trial trucks. The control group served as a contextual benchmark to support the observed outcomes in the trial group, rather than as a strictly matched comparator.

To further improve the proposed protocol, it is recommended to ensure that surfaces are completely dry before applying the disinfectant, as excessive water accumulation inside the vehicle can dilute the disinfectant and compromise its effectiveness, while increased residual moisture may also favor bacterial survival and proliferation (21). In addition, the improved protocol should also include cleaning both underneath and on top of the truck. Further research should focus on the validation of this C&D protocol in the field, and on individual ATP threshold and critical limits. To ensure the protocol's effectiveness and long-term adoption, it should ideally be supported by regular training and reinforced through oversight by competent authorities or independent control bodies. To improve biosecurity of trucks, other strategies may be used, such as rerouting, as proposed by Galvis et al. (65, 69). Finally, the authors emphasize that improving truck hygiene does not justify non-compliance with haulers' biosecurity measures. Although the responsibility of the truck C&D lies with the animal transport driver (5), the widespread use of contract livestock haulers (instead of farmed-owned transport) hinders the enforcement and monitoring of biosecurity compliance (3). Haulers must remain aware of proper biosecurity practices on farms to avoid undermining the efforts invested in C&D.”

The references were listed incorrectly in this order:

60. Sawodny S, Käsbohrer A, Bröker L, Firth C, Marschik T. Intervention strategies for methicillin-resistant Staphylococcus aureus control in pig farming: a comprehensive review. Porc Health Manag. (2025) 11:17. doi: https://doi.org/10.1186/s40813-025-00435-8.

61. Broens EM, Espinosa-Gongora C, Graat EA, Vendrig N, Van Der Wolf PJ, Guardabassi L, et al. Longitudinal study on transmission of MRSA CC398 within pig herds. BMC Vet Res. (2012) 8:58. doi: https://doi.org/10.1186/1746-6148-8-58.

62. Merialdi G., Galletti E., Rugna G., Granito G., Franco A., Battisti A., et al. Longitudinal study on methicillin resistant Staphylococcus aureus (MRSA) nasal colonization in a farrow to finish pig herd. In: Proceedings of the 4th European symposium of porcine health management; 2012 Apr 25–27; Bruges, Belgium. (2012). p. 100. 63.

63. Beslay C, Bouix M, Fauroux H, Gautier A, Luposella F, Pillon J-M, et al. ≪Un lavage, c'est un lavage≫. Des chauffeurs bovins face aux consignes sanitaires. Les Mondes du Trav ai l. (2023) 29:147–63. 64.

64. Moormeier DE, Bayles KW. Staphylococcus aureus biofilm: a complex developmental organism. Mol Microbiol. (2017) 104:365–76. doi: 10.1017/S0950268810000245.

65. Galvis JA, Corzo CA, Machado G. Mitigating between-farm disease transmis-sion through simulating vehicle rerouting and enhanced cleaning and disinfection protocols. Prev Vet Med. (2025) 244:106650. doi: 10.1016/j.prevetmed.2025.106650.

66. Dierikx CM, Hengeveld PD, Veldman KT, de Haan A, van der Voorde S, Dop PY, et al. Ten years later: still a high prevalence of MRSA in slaughter pigs despite a signifi-cant reduction in antimicrobial usage in pigs the Netherlands. J Antimicrob Chemother. (2016) 71:2414–8. doi: 10.1093/jac/dkw190.

67. Mulders MN, Haenen AP, Geenen PL, Vesseur PC, Poldervaart ES, Bosch T, et al. Prevalence of livestock-associated MRSA in broiler flocks and risk factors for slaughterhouse personnel in the Netherlands. Epidemiol Infect. (2010) 138:743–55. doi: 10.1017/S0950268810000075.

68. Ingham AC, Urth TR, Sieber RN, Stegger M, Edslev SM, Angen Ø, et al. Dynamics of the human nasal microbiota and Staphylococcus aureus CC398 carriage in pig truck drivers across one workweek. Appl Environ Microbiol. (2021) 87:e0122521. doi: 10.1128/AEM.01225-21.

69. Van Cleef BA, Broens EM, Voss A, Huijsdens XW, Züchner L, Van Benthem BH, et al. High prevalence of nasal MRSA carriage in slaughterhouse workers in contact with live pigs in the Netherlands. Epidemiol Infect. (2010) 138:756–63. doi: 10.1017/S0950268810000245.

The relevant in-text citations have been corrected in section **4.2 Trucks C&D effectiveness against MRSA**, paragraphs 6-10, as follows:

The high prevalence of MRSA even after C&D has been highlighted as a critical One Health issue (61), since MRSA is considered an indicator organism for the presence of antibiotic-resistant bacteria for both animals and humans (54). In this context, while the transmission of MRSA from truck drivers to pigs has not been conclusively demonstrated (59), available data suggest that drivers are more likely to be victims of occupational exposure than sources of infection. Studies in pig and poultry abattoirs have reported MRSA prevalences ranging from 22% to 40% among lorry drivers (62–64), underscoring the importance of robust hygiene protocols, not only to limit environmental MRSA loads and protect animal health, but also to safeguard the health of transport workers operating in high-risk settings.

Searching for the best application of a C&D protocol, the proper selection of disinfectants should not be overlooked, as their documented efficacy against priority swine pathogens, including African swine fever virus (8), as well as their ability to act against bacterial biofilms, can ultimately determine the success of C&D. MRSA, for example, is a biofilm-producing pathogen, recognized for its persistence and capacity to cause chronic infections, including in hospitals, where biofilm formation confers additional resistance to C&D (65). However, the available literature indicates that biofilm formation represents only one of several mechanisms potentially involved in MRSA persistence. MRSA persistence in livestock production systems is widely acknowledged to be multifactorial, resulting from the interaction between microbial characteristics, environmental contamination, animal-related factors, and management practices. In swine production, Merialdi et al. (50) demonstrated a preventive effect of C&D procedures on environmental contamination, although this effect was not constant across productive phases and was more pronounced in farrowing crates than in later stages. Longitudinal studies (66, 67) have shown that MRSA nasal carriage dynamics vary markedly with age, with differences in prevalence among herds largely attributed to management practices, while the role of the environment has been recognized as contributory and deserving further investigation.

The time required for the complete and proper execution of the developed hygiene protocol can be considered among the factors contributing to the improved sanitary status of the trial trucks, as it is longer than the average time recorded for control trucks at the slaughterhouse. Indeed, time constraints are frequently reported as a reason for haulers' non-compliance with truck C&D procedures (5, 15, 68). While a duration of two and a half hours may be feasible for trucks transporting live pigs, it is hardly compatible with the slaughterhouse's tight routine. Nevertheless, this protocol could serve as a starting point for improving hygiene management in market trucks, tailoring it to fit the slaughterhouse's tight schedule while still ensuring an adequate sanitary level.

A limitation of this study is that the trial and control trucks did not belong to the same production chain and therefore transported different groups of pigs, which may have varied in terms of age, origin and health status. These differences could have influenced the baseline microbial load, particularly in compartments such as the interior cargo area. However, it is important to note that the aim of the study was not to directly compare performance between different fleets, but rather to demonstrate the effectiveness of the standardized C&D protocol applied to the trial trucks. The control group served as a contextual benchmark to support the observed outcomes in the trial group, rather than as a strictly matched comparator.

To further improve the proposed protocol, it is recommended to ensure that surfaces are completely dry before applying the disinfectant, as excessive water accumulation inside the vehicle can dilute the disinfectant and compromise its effectiveness, while increased residual moisture may also favor bacterial survival and proliferation (21). In addition, the improved protocol should also include cleaning both underneath and on top of the truck. Further research should focus on the validation of this C&D protocol in the field, and on individual ATP threshold and critical limits. To ensure the protocol's effectiveness and long-term adoption, it should ideally be supported by regular training and reinforced through oversight by competent authorities or independent control bodies. To improve biosecurity of trucks, other strategies may be used, such as rerouting, as proposed by Galvis et al. (69). Finally, the authors emphasize that improving truck hygiene does not justify non-compliance with haulers' biosecurity measures. Although the responsibility of the truck C&D lies with the animal transport driver (5), the widespread use of contract livestock haulers (instead of farmed-owned transport) hinders the enforcement and monitoring of biosecurity compliance (3). Haulers must remain aware of proper biosecurity practices on farms to avoid undermining the efforts invested in C&D.

References have been re-order as follows:

60. Dierikx CM, Hengeveld PD, Veldman KT, de Haan A, van der Voorde S, Dop PY, et al. Ten years later: still a high prevalence of MRSA in slaughter pigs despite a significant reduction in antimicrobial usage in pigs the Netherlands. *J Antimicrob Chemother*. (2016) 71:2414–8. doi: 10.1093/jac/dkw190.

61. Sawodny S, Käsbohrer A, Bröker L, Firth C, Marschik T. Intervention strategies for methicillin-resistant *Staphylococcus aureus* control in pig farming: a comprehensive review. *Porc Health Manag*. (2025) 11:17. doi: 10.1186/s40813-025-00435-8.

62. Mulders MN, Haenen AP, Geenen PL, Vesseur PC, Poldervaart ES, Bosch T, et al. Prevalence of livestock-associated MRSA in broiler flocks and risk factors for slaughterhouse personnel in the Netherlands. *Epidemiol Infect*. (2010) 138:743–55. doi: 10.1017/S0950268810000075.

63. Ingham AC, Urth TR, Sieber RN, Stegger M, Edslev SM, Angen Ø, et al. Dynamics of the human nasal microbiota and *Staphylococcus aureus* CC398 carriage in pig truck drivers across one workweek. *Appl Environ Microbiol*. (2021) 87:e01225–21. doi: 10.1128/AEM.01225-21.

64. Van Cleef BA, Broens EM, Voss A, Huijsdens XW, Züchner L, Van Benthem BH, et al. High prevalence of nasal MRSA carriage in slaughterhouse workers in contact with live pigs in The Netherlands. *Epidemiol Infect*. (2010) 138:756–63. doi: 10.1017/S0950268810000245.

65. Moormeier DE, Bayles KW. *Staphylococcus aureus* biofilm: a complex developmental organism. *Mol Microbiol*. (2017) 104:365–76. doi: 10.1111/mmi.13634.

66. Broens EM, Espinosa-Gongora C, Graat EA, Vendrig N, Van Der Wolf PJ, Guardabassi L, et al. Longitudinal study on transmission of MRSA CC398 within pig herds. *BMC Vet Res*. (2012) 8:58. doi: 10.1186/1746-6148-8-58.

67. Merialdi G, Galletti E, Rugna G, Granito G, Franco A, Battisti A, et al. Longitudinal study on methicillin resistant *Staphylococcus aureus* (MRSA) nasal colonization in a farrow to finish pig herd. In: *Proceedings of the 4th European Symposium of Porcine Health Management*. Bruges (2012). p. 100.

68. Beslay C, Bouix M, Fauroux H, Gautier A, Luposella F, Pillon J-M, et al. ≪Un lavage, c'est un lavage≫. Des chauffeurs bovins face aux consignes sanitaires. *Les Mondes du Travail*. (2023) 29:147–63.

69. Galvis JA, Corzo CA, Machado G. Mitigating between-farm disease transmission through simulating vehicle rerouting and enhanced cleaning and disinfection protocols. *Prev Vet Med*. (2025) 244:106650. doi: 10.1016/j.prevetmed.2025.106650.

The original article has been updated.

