# Supplementary material for: Hygiene procedures of trucks transporting live pigs: multi-assessment validation of a standardized C&D protocol
Source: Front Vet Sci. 2026 Mar 19;13:1789484. doi: 10.3389/fvets.2026.1789484 (PMC13045574; doi:10.3389/fvets.2026.1789484)
Supplement: Supplementary file 1 [file Table_1.docx]

Supplementary Material

## Checklist for visual evaluation of cleaning and disinfection procedures of trucks transporting live pigs

User guide

Rate 1:  unsatisfactory cleaning, more than 85% of the surface is contaminated, or where gross, highly evident contamination is visible.

Rate 2: discrete cleaning, only traces of organic matter are present (covering less than 15% of the surface)

Rate 3: perfect cleaning, no visible organic matter.

**Inspector:                          Date:                          Time:                          Truck:**

|  | **Item** | **RATE (1-2-3)** | **NOTES** |
| --- | --- | --- | --- |
|  | **Boot storage** |  |  |
| 1 | Internal surfaces |  |  |
| 2 | External surface |  |  |
| 3 | Handle |  |  |
| 4 | Boots |  |  |
|  | **Exterior surface of the truck** |  |  |
| 5 | Wheels |  |  |
| 6 | Truck lateral axes |  |  |
| 7 | Trailer external surface |  |  |
| 8 | Tractor external surface |  |  |
| 9 | Rearview mirrors |  |  |
| 10 | Footstep |  |  |
| 11 | License plate |  |  |
| 12 | Loading ramp - exterior side |  |  |
| 13 | Tractor-trailer connection part |  |  |
| 14 | Tractor windows |  |  |
|  | **Inside cargo area** |  |  |
| 15 | Floor |  |  |
| 16 | Right side wall |  |  |
| 17 | Left side wall |  |  |
| 18 | Front wall |  |  |
| 19 | Ceiling |  |  |
| 20 | Movable partitions |  |  |
| 21 | 4 upper corners |  |  |
| 22 | 4 lower corners |  |  |
| 23 | Air fans |  |  |
| 24 | Sliding chains |  |  |
| 25 | Lateral grilles |  |  |
| 26 | Nipple drinkers |  |  |
| 27 | Loading ramp - inside surface |  |  |
| 28 | Rear door closing/opening systems |  |  |
|  | **Driver’s cab** |  |  |
| 29 | Steering wheel |  |  |
| 30 | Pedals |  |  |
| 31 | Seats |  |  |
| 32 | Floor |  |  |
| 33 | Windows - inside surface |  |  |
| 34 | Mats |  |  |
